# Flexibility of movement organization in piano performance

**DOI:** 10.3389/fnhum.2013.00173

**Published:** 2013-07-16

**Authors:** Shinichi Furuya, Eckart Altenmüller

**Affiliations:** Institute for Music Physiology and Musician's Medicine, Hannover University of Music, Drama and MediaHannover, Germany

**Keywords:** degrees of freedom, fine motor control, redundancy, optimal control, focal dystonia

## Abstract

Piano performance involves a large repertoire of highly skilled movements. The acquisition of these exceptional skills despite innate neural and biomechanical constraints requires a sophisticated interaction between plasticity of the neural system and organization of a redundant number of degrees of freedom (DOF) in the motor system. Neuroplasticity subserving virtuosity of pianists has been documented in neuroimaging studies investigating effects of long-term piano training on structure and function of the cortical and subcortical regions. By contrast, recent behavioral studies have advanced the understanding of neuromuscular strategies and biomechanical principles behind the movement organization that enables skilled piano performance. Here we review the motor control and biomechanics literature, introducing the importance of describing motor behaviors not only for understanding mechanisms responsible for skillful motor actions in piano playing, but also for advancing diagnosis and rehabilitation of movement disorders caused by extensive piano practice.

## Introduction

**Figure 1:**
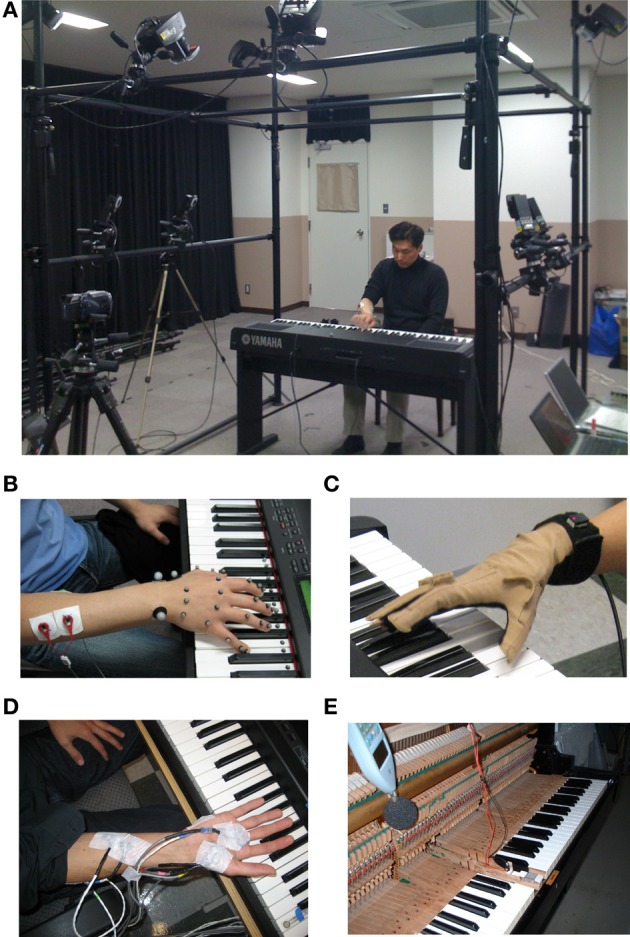
**Devises for behavioral measurements. (A)** Motion capture system with high speed cameras. **(B)** Reflective markers for the motion capture. **(C)** Data glove. **(D)** Surface electromyography. **(E)** Force sensor embedded on the surface of a piano key.

Outstanding musical performance has fascinated people over centuries. It is built on exceptional sensory, cognitive, and motor abilities, which include fast, accurate, dexterous, and efficient movements, production of rich repertoires of complex motions, quick correction of erroneous actions, sensory-motor coordination, and memory that stores vast musical repertoires and is recalled quickly. In the past years, researchers have attempted to clarify the neural mechanisms and neuroplasticity subserving the virtuosity of musicians by using neuroimaging techniques, such as fMRI, PET, MEG, and EEG. These “top–down” studies have demonstrated functional and structural neuroplastic changes at cortical and subcortical regions associated with sensory, cognitive, and motor abilities (Münte et al., 2002; Zatorre et al., 2007; Jäncke, 2009; Wan and Schlaug, 2010; Pantev and Herholz, 2011; Herholz and Zatorre, 2012). Superior perceptual and cognitive abilities of musicians were also addressed behaviorally (Ragert et al., 2004; Stewart et al., 2004). However, up to now, only few studies were devoted to behavioral features of distinguished motor skills of musicians. Methodologically, behavioral studies of motor skill include measurements of movements using motion-capture, electromyography, and force sensors (Figure 1), and data analysis including computational analysis such as robotics, signal processing, multivariate analysis, and machine learning. This line of “bottom–up” study, often called *reverse engineering* approach, provides a unique opportunity of inferring neural strategies and biomechanical principles underlying the production of virtuosic motor performance of musicians. In this review, we focus on behavioral studies that probed motor control and learning of skilled piano performance in order to better understand the mechanisms of accomplished musical performance. Of specific interest is the distinct organization of the redundant number of degrees of freedom (DOFs) in the upper limb allowing the production of fast, accurate, and efficient piano performance by expert players. Furthermore, as an example of maladaptive neuroplasticity, we briefly mention focal dystonia in pianists, a neurological disorder characterized by a degradation of fine motor control of highly overlearned skilled movements.

## Reorganization of redundant multi-joint arm movements in piano keystrokes

The motor system has a redundant number of joints and muscles (DOFs) (Bernstein, 1967). This indicates that the same movement can be performed through a multitude of different combinations of individual joint movements and muscular activities. In piano performance, for example, this redundancy allows for the production of a certain acoustic event with various ways of organization of the DOFs. As a result of practice, neuroplasticity leads to the reorganization of the neuromuscular system, which in turn yields improvements of skilled motor action. A common approach to better understand the interaction between neuroplasticity, motor redundancy, and organization principles governing graceful motor behaviors in piano performance is to describe differences in the movement organization between skilled and unskilled individuals. This approach uniquely allows for inferring the effect of long-term training on the movement organization, which is hard to experimentally assess through longitudinal studies.

**Figure 2:**
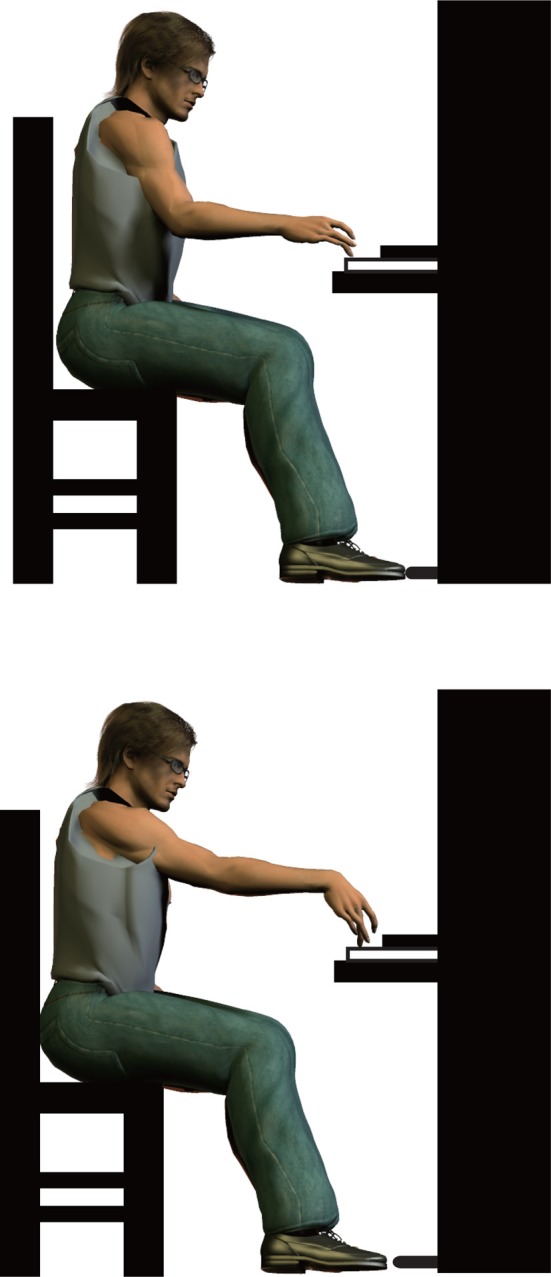
**An example that describes the kinematic redundancy**. A fingertip motion cannot uniquely specify the joint kinematics due to the redundancy of the upper-limb.

The motor system of pianists as an example has several levels of redundancy for the production of a tone. First, a target tone can be elicited by an infinite number of possible spatio-temporal profiles of force and movement at the fingertip (endpoint redundancy). Second, a given motion at the fingertip can emerge as a consequence of a variety of possible spatio-temporal coordinative movements across multiple joints (kinematic redundancy). Third, a given rotation at a joint can be generated by complex interactions of different joint torques (i.e., rotational force) originating from muscular, gravitational, inter-segmental and reaction forces (kinetic redundancy). Fourth, muscular torque emerges as a balance of forces generated by agonist and antagonist muscles surrounding a joint (muscular redundancy). The redundancy of the motor system therefore provides infinite possible ways of organizing the upper limb movements even for a single tone production (Figure 2).

The problem of endpoint redundancy indicates that loudness and duration of a single tone cannot define a unique waveform of the fingertip force. For instance, both impulsive force production via hitting a key and progressive force production during depressing a key can elicit the same target velocity of a key (Kinoshita et al., 2007). Similarly, the target duration of a tone requires continuous production of force that amounts above the minimum force that prevents a key from lifting up. Interestingly, the amount of the force to keep a key depressed was larger for the recreational pianists than the experts, although duration of the elicited tone was the same (Parlitz et al., 1998). The smaller amount of residual force suggests a distinct solution of the endpoint redundancy in a tone production by the skilled pianists so as to economize energy expenditure. Overall, this confirms that a musical sound with the same loudness and duration can be generated through a multitude of possible forces.

The kinematic redundancy allows for flexible organization of multi-joint movements (Yang and Scholz, 2005). During the production of a piano tone, the fingertip motion originates primarily from rotation of the shoulder, elbow, wrist, and finger joints. To probe flexibility of the organization of multi-joint movements in a piano keystroke, several studies characterized spatio-temporal features of these multi-joint motions in skilled and unskilled musicians. During alternate keystrokes with the thumb and little finger (i.e., tremolo) at a particular tempo and loudness, skilled pianists elicited faster elbow pronation-supination rotation and slower finger rotation compared to unskilled players (Furuya et al., 2011b). The experts also exerted a smaller amount of co-activation at the extrinsic finger muscles. These findings suggested improved efficiency of the distal muscles prone to fatigue via kinematic reorganization that takes advantage of the proximal joint motion in piano keystrokes.

Because joint kinematics (i.e., motion and posture) affect joint kinetics (i.e., torques), kinematic and kinetic redundancy problems are to some extent associated. During simultaneous keystrokes with the thumb and little finger (i.e., octave) at a certain loudness, the arm downswing motion was characterized by a sequence of joint rotations in an order from proximal to distal for the expert pianists, but not for the novice players (Furuya and Kinoshita, 2007). This sequencing motion is typically observed in skilled motor behaviors such as throwing and kicking, and serves as a mechanism to accelerate the endpoint of the limb effectively (Putnam, 1993). The proximal-to-distal sequence creates deceleration of the proximal joint rotation during the period in which the distal joint is accelerating. The proximal joint's deceleration generates the inter-segmental dynamics that drive the distal joint rotation (Hirashima and Ohtsuki, 2008). This phenomenon can be directly assessed by the inverse dynamics technique that computes joint rotational force (torque) based on information of movements and force. It decomposes net joint torque into constituent torques that originates from gravity, inter-segmental dynamics, muscular contraction, and mechanical interactive force. The inverse dynamics have therefore provided insights into neural control of multi-joint arm movements (Hollerbach and Flash, 1982; Bagesteiro and Sainburg, 2002; Hirashima et al., 2007; Dounskaia, 2010). In the piano keystroke, the expert pianists produced larger inter-segmental dynamics and smaller muscular torque at the elbow and wrist joints during hand downswing than the novice players (Furuya and Kinoshita, 2008a). This finding, in combination with the kinematic observations, indicates that the distinct temporal coordination of the joint rotation yielded the distinct coupling between muscular and non-muscular forces in the keystroke by skilled and unskilled pianists, providing the former individuals with superior “physiological” efficiency.

The associations of kinematic and kinetic redundancy problems are also evident while the fingertip was contacting with the piano key. In this case, the force of the fingertip is counteracted by the reaction force from the key, according to an action-reaction principle by Newtonian physics. Because this reaction force from the key generates joint torque that impedes the key-depressing motion (i.e., reaction-force torque), muscles need to generate a joint torque that counteracts with this interfering dynamics (Harding et al., 1993). The reaction-force torque varies in relation to both the magnitude of force and geometric configuration of the upper-limb (i.e., posture). Production of a piano tone at particular loudness therefore yields different reaction-force torques depending on the limb posture. Indeed, the limb posture is a key variable for successful compensation for the mechanical interaction with external dynamics (Lacquaniti et al., 1992). During the depression of a piano key, the expert pianists rotated the shoulder joint for flexion and thereby configured an upright posture of the finger, which, in contrast was not evident for novice players (Furuya and Kinoshita, 2008b). This postural configuration lowers the reaction-force torque at the finger joint, the finger muscular torque, and the finger extrinsic muscular activity, which again indicates the interaction between the kinematic and kinetic organization in a way of providing more skilled individuals with a posture with smaller mechanical perturbation and muscular work (Furuya and Kinoshita, 2008a,b).

The muscular redundancy emerges due to multiple muscles crossing a joint. Production of a certain amount of muscular torque can be therefore achieved by different combinations of forces across muscles. The simplest example is the agonist and antagonist muscles during the elbow extension in the vertical plane. The elbow joint rotates for extension by either contracting the extensor muscle or relaxing the anti-gravity flexor muscle to utilize gravity. Indeed, the recording of arm muscular activity demonstrated that production of the elbow extension muscular torque during the arm descent in piano keystrokes was associated with an increase in extensor muscular activity for novice players, and with a decrease in flexor muscular activity for expert piano players, respectively (Furuya et al., 2009). This finding indicates distinct solutions to the muscular redundancy problem depending on levels of proficiency of pianists so that the long-term piano training can achieve physiological efficiency by utilizing gravity during the piano keystroke.

In sum, the cross-sectional studies that compared skilled and unskilled pianists have provided converging evidence supporting for skill-level dependent organization of the upper limb movements so as to facilitate physiological efficiency following extensive piano training. This idea is in agreement with empirical findings of learning-dependent minimization of physiological cost in movements such as reaching (Thoroughman and Shadmehr, 1999; Osu et al., 2002; Huang et al., 2012) and walking (Finley et al., 2013), and with the theoretical framework of minimization of muscular fatigue in well-learned tasks (Prilutsky and Zatsiorsky, 2002). Furthermore, repetitive and forceful piano keystrokes for 30 min without fatiguing muscles were possible only for the skilled pianists but not for unskilled individuals (Furuya and Kinoshita, 2008a). This finding confirms that the specialized movement organization acquired through extensive piano training enables to circumvent muscular fatigue and maintain high levels of excellence in music performance. This fits with the idea of optimal control that optimizes task-dependent cost functions to specify a movement (Flash and Hogan, 1985; Soechting et al., 1995; Harris and Wolpert, 1998; Todorov and Jordan, 2002).

## Hand motor control in piano playing

The human hand can be conceived as a motor system with a large number of DOF, comprising in total 27 bones and 36 muscles. These effectors enable the production of fast and dexterous motor behaviors such as grasping, typing, finger spelling, surgery, and musical performance. A key issue in neural control of hand movements is how the nervous system utilizes the DOFs to produce rich repertoires of dexterous motor actions. In any hand movements, at least two distinct patterns of finger joint coordination are evident; coupled and individuated movements across fingers. The coupled motions represent covariation of joint motion across fingers. For example, two covariation patterns of the finger movements, which represented power and precision grips, described hand motions during grasping objects with various shapes and sizes (Santello et al., 1998, 2002; Mason et al., 2001; Ingram et al., 2008; Thakur et al., 2008). The coupled finger movements were also evident during the thumb keystroke in piano playing (Furuya et al., 2011a). Principal component analysis and cluster analysis for the hand kinematics identified two distinct covariation patterns of movements, which described the thumb keystroke during playing over 60 different tone sequences. Remarkably, both of these two patterns in common displayed simultaneous motion across fingers, forming the coupled finger motions. Repetitive use of the coupled finger motions in music performance may facilitate finger coordination and movement accuracy of motor tasks irrelevant to piano playing such as grasping (Fernandes and De Barros, 2012), possibly due to a decrease of surround inhibition across hand muscles (Shin et al., 2012). In addition, these two patterns of hand motion differed in timing of thumb rotation depending on whether the hand opens or closes before and after the thumb keystroke, which suggests independent use of the thumb from the fingers.

Individuated finger movements, in which one or more fingers are moved relatively independently of the movement or posture of other fingers (Schieber, 1995), play a key role in the dexterous use of the hand, such as configuration of complex hand shape and production of precisely timed sequences of movements (Fuglevand, 2011; Van Duinen and Gandevia, 2011). In piano performance, for keystrokes with each of the four fingers during playing various tone sequences, the hand kinematics was characterized by three distinct patterns of finger joint coordination (Furuya et al., 2011a). The motion of the striking finger was consistent across these patterns, whereas the motion of the non-striking fingers differed across them. This was interpreted as evidence for the independence of movements across fingers. In addition, the amount of movement covariation between the striking and non-striking fingers was similar, independent of which finger was used for a keystroke. The finding was in contrast to non-musicians who displayed a hierarchy of independence of finger movements, the middle and ring fingers being less individuated than the index and little fingers (Häger-Ross and Schieber, 2000; Zatsiorsky et al., 2000). The equal independence of movements across fingers can be therefore achieved by extensive piano training. This idea is supported by superior independence of finger movement control for pianists as compared to non-musicians (Slobounov et al., 2002; Aoki et al., 2005), which possibly occurs due to changes at biomechanical and neural levels (Chiang et al., 2004; Smahel and Klimová, 2004). Early piano training can also facilitate the robustness of the motor skills enabling individuated finger movements in expert pianists, as became evident from a recent study applying transcranial direct current stimulation (tDCS) over the motor cortex in pianists: pianists with an older age at inception of piano practice showed a more pronounced effect of motor cortex stimulation,—i.e., increase in speed and accuracy of finger movements, as compared to pianists who commenced piano training earlier (Furuya et al., 2013).

Plasticity of the representations of dexterous finger movements at the central nervous system can be addressed by using non-invasive transcranial magnetic stimulation (TMS). A comparison of hand movements elicited by TMS over the primary motor cortex between pianists, violinists and non-musicians identified distinct movement features associated with the trained movement repertoire (Gentner et al., 2010). This observation provided evidence for encoding of experience-dependent motor skills in the functional organization of the primary motor cortex and its efferent system. Furthermore, linear combinations of a selected subset of joint correlation patterns in TMS-evoked finger movements successfully reconstructed movement features during the trained motor behaviors (i.e., playing piano and violin for pianists and violinists, respectively). This finding, together with the behavioral observation of a small number of fundamental movement patterns in piano playing (Furuya et al., 2011a), suggests a simplification in organization of multiple DOFs of the hand. This can be a common neural mechanism in order to simplify hand motor control across various motor repertoires (Santello et al., 2002; Hart and Giszter, 2004; D'avella and Bizzi, 2005; Gentner and Classen, 2006; Overduin et al., 2012).

In piano performance, not all digits necessarily move for the production of a tone. Depending on contexts and task demands, some digits either move anticipatorily to facilitate production of upcoming acoustic events or even do not have to move. The former anticipatory modification of the movements is called coarticulation and serves as a mechanism that ensures smooth succession of sequential movements such as speech (Ostry et al., 1996) and finger spelling (Jerde et al., 2003). This coarticulation was also evident in piano playing, particularly when the hand posture changes dynamically (Engel et al., 1997). For example, the fingers and wrist initiated preparatory motions 500 ms prior to the thumb-under maneuver, which facilitated the subsequent horizontal translation of the hand. Finger muscular activity also provided evidence supportive for co-articulation in piano playing (Winges et al., 2013). The balance of burst amplitudes across multiple muscles depended on the characteristics of the preceding and subsequent keypresses, forming neuromuscular co-articulation throughout the time course of sequential finger movements.

When some digits do not have to move for tone production, they form a certain posture by static muscular contraction. A posture can be seen as the equilibrium point defined by the balance of forces between flexor and extensor muscles (Ostry and Feldman, 2003). Therefore, the posture of digits being not used for keystrokes changes in relation to the static force exerted by the respective finger muscles. Interestingly, the posture of task-irrelevant digits differed between skilled and unskilled pianists during alternate keystrokes with the thumb and little finger (Furuya et al., 2011b). Pianists with superior skill displayed smaller extension angles at the index and middle fingers over various tempi, and also smaller activity of the extrinsic finger muscles. Expertise-dependent reorganization of the posture of task-irrelevant digits was therefore likely to facilitate physiological efficiency.

## Manipulation of elements of music

Expressive musical performance may require sensorimotor skills that enable pianists to manipulate various elements of music (e.g., loudness, tempo, timbre, rhythm). This involves not only parametric modulation of spatial and temporal features of movements, but also movement reorganization of the motor system. Plasticity of the nervous system allows through extensive piano training the optimization of movement control involved in adjustment of the individual elements of music. For example, to manipulate the loudness of a piano tone, skilled pianists and unskilled individuals reorganized the upper limb movements and muscular coordination in a distinct manner. In order to increase the velocity of elbow rotation during the hand downswing for a louder tone production, the experts elicited larger inter-segmental dynamics by increasing the amount of the shoulder joint deceleration, whereas the novices simply generated lager elbow muscular torque (Furuya and Kinoshita, 2007, 2008a). The loudness increase was also associated with a decrease in the anti-gravity activity of the elbow flexor and increase in the activity of the elbow extensor for the expert pianists and novice players, respectively (Furuya et al., 2009). These findings suggest effects of extensive long-term piano training on the movement organization and muscular coordination responsible for loudness control, yielding larger reliance on non-muscular forces.

Tempo control also influences the movement organization distinctly between skilled and unskilled players. During alternate keystrokes with the thumb and little finger (i.e., tremolo), an increase in tempo yielded increases in rotational velocity at both elbow and finger (Furuya et al., 2011b). Interestingly, expert pianists showed smaller increase at the finger and larger increase at the elbow than amateur pianists, resulting in the more effective use of proximal muscles with greater endurance to fatigue. When expert pianists were playing musical pieces, the joint kinematics of the fingers did not differ between the normal tempo (8 strokes/s) and fast tempo (11.5 strokes/s) (Furuya and Soechting, 2012), which was the case even among a wider range of tempi (Goebl and Palmer, 2013). Furthermore, the timing accuracy of keystrokes was also maintained across tempi (Furuya and Soechting, 2012; Goebl and Palmer, 2013), which violated the speed-accuracy tradeoff (Fitts, 1954). These tempo-invariant finger kinematics were in contrast to observations in musically-naïve individuals who displayed larger covariation of joint motions across fingers when moving a finger faster (Häger-Ross and Schieber, 2000). Taken together, the effect of tempo adjustment on movement organization differed between the skilled and unskilled piano players, providing more skilled pianists with superior physiological efficiency and independent control of finger movements.

Variations of the timbre of a piano tone also play a role in expressive musical performance. Perception of timbre varies with noises that emerge from the collisions between the fingertip and the key surface (touch noise) and between the key and key-bed (bottom noise) (Goebl et al., 2004; Goebl and Fujinaga, 2008). The mechanical noises change with the way of touching a piano key; for example, a key-depression either with or without preparatory lift of the finger, elicits a tone with hard and soft timbre, respectively (Furuya et al., 2010). These touches were associated with different patterns of joint coordination and inter-segmental dynamics (Furuya et al., 2010). The former and latter touch involved the proximal-to-distal and distal-to-proximal sequencing joint rotations, and generated the inter-segmental dynamics that accelerated the distal and proximal joints, respectively. The manipulation of kinematic and kinetic features of the upper limb movements could thus allow for variations in tone timbre. In light of this, the distinct solution of endpoint redundancy of pianists with different level of proficiency can be associated with differences in timbre of a tone to be produced. It is also possible that different somatosensory feedback between the two touches plays a role in the timbre manipulation (Goebl and Palmer, 2008).

## Individual differences of movements across pianists

The hand and arm movements in piano performance differ even across skilled pianists. The individual differences can manifest themselves from numerous intrinsic and extrinsic factors including anatomical and physiological properties of the musculoskeletal system, structure and function of the nervous system, practice regime, history of education, and neural and biomechanical strategies. Neuroimaging studies demonstrated that the structure of cortical and subcortical regions predicted speed and accuracy of skilled finger movements in individual players (Amunts et al., 1997; Granert et al., 2011). Recently, several behavioral studies addressed the individual differences in movement kinematics and muscular activities across pianists (Dalla Bella and Palmer, 2011; Furuya et al., 2011b, 2012; Goebl and Palmer, 2013). A motion capture study with four skilled pianists revealed that information that identifies individual pianists was encoded in kinematic features of the fingertip movements during piano playing (Dalla Bella and Palmer, 2011). In 18 skilled pianists, the variations of velocities at the shoulder, elbow, wrist and finger joints in relation to loudness and tempo during repetitive keystrokes could be categorized into three groups according to distinct joint coordination (Furuya et al., 2012). Muscular load also differed across these groups, which implicates a potential of addressing individual differences in the movements for predicting risk factors of playing-related injuries. During fast alternate keystrokes with the thumb and little finger(tremolo), the maximum rate of keystrokes was correlated with the maximum elbow velocity but not with the finger velocity across 10 pianists, which highlights the importance of proximal joint motions for fast piano performance (Furuya et al., 2011b). A recent study that investigated the hand kinematics while 12 pianists were playing a simple tone sequence identified a correlation between a measure that represents the finger joint coordination and timing accuracy and precision of keystrokes of individual pianists (Goebl and Palmer, 2013). It is therefore likely that individual differences in movement organization also are reflected in the quality of performance at least with respect to the parameters mentioned above.

## Auditory-motor integration

Musicians have a neural mechanism that integrates auditory and motor information (Bangert and Altenmüller, 2003; Bangert et al., 2006; D'ausilio et al., 2006; Baumann et al., 2007; Lahav et al., 2007; Luo et al., 2012; Stewart et al., 2013). Behavioral studies demonstrated that auditory information modulates the movement organization in music performance in both feedforward and feedback manners (Keller, 2012; Pfordresher, 2012). In a sequential tone production task with a keyboard, the fingertip motion displayed greater acceleration prior to collision with the key when the key locations were spatially incompatible with pitch as compared to when they were compatible (Keller et al., 2010). The observation of this phenomenon prior to the first stroke suggests that anticipatory auditory imagery modulates the spatial and temporal features of movement organization in regularly timed auditory action sequences. In addition, the influence of anticipatory auditory imagery of movement production seems more salient for musicians than non-musicians, suggesting an effect of musical training (Keller and Koch, 2008). By contrast, erroneous keystrokes, which are typically caused by pitch alteration during piano playing (Pfordresher, 2003; Furuya and Soechting, 2010), occurred more frequently for pianists than non-musicians (Pfordresher, 2005), implying more reliance on auditory feedback in more skilled pianists [however, the opposite finding was also reported (Pfordresher, 2012)].

## Focal hand dystonia

**Figure 3:**
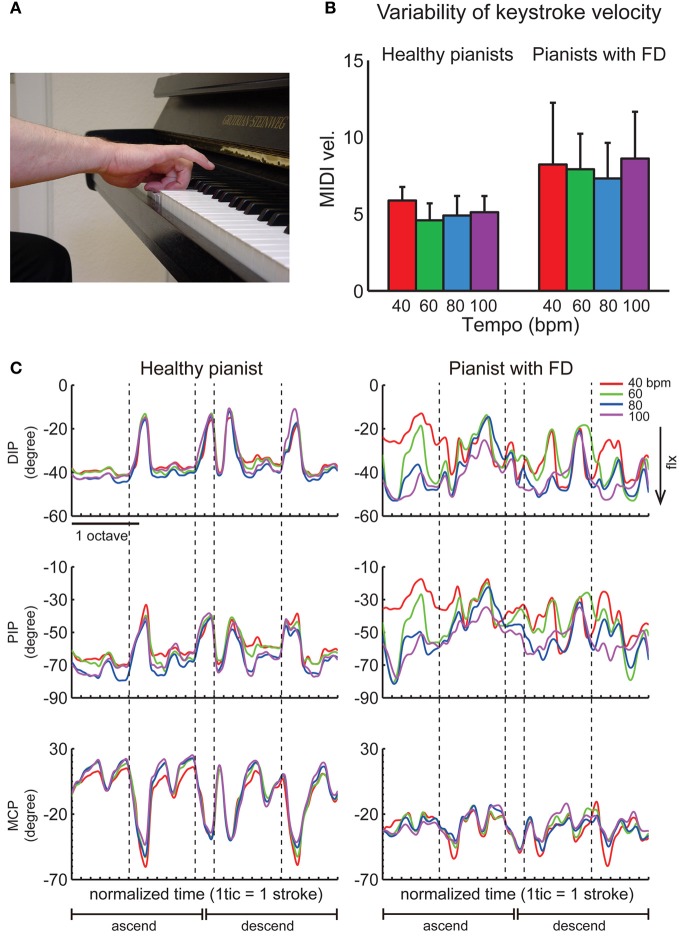
**(A)** A typical symptom of an involuntary hyper-flexion of a pianist with focal dystonia. **(B)** A group mean of variability of the keystroke velocity across 10 healthy pianists (left) and 10 pianists with focal dystonia (right) during playing a C-major scale with the right hand at four different tempi [40, 60, 80, and 100 beat per minute (BPM)]. **(C)** The time-varying waveforms of the angles at the distal-interphalangeal (DIP), proximal-interphalangeal (PIP), and metacarpo-phalangeal (MCP) joints of the ring finger of a healthy pianist (left panel) and a pianist having the ring finger affected (right panel) during playing a two-octave C-major scale in both ascending and descending directions at four different tempi (different colors). Each tick indicates the moment of each keystroke, and each vertical dotted line indicates the moment of a stroke with the ring finger. The negative value defines joint flexion. The joint angle was measured by a custom-made data glove (Gentner and Classen, 2009). **(B,C)** are derived from Furuya and Altenmüller (2012).

Focal hand dystonia is a neurological disorder characterized by involuntary movements, twisting, abnormal postures and co-contraction of antagonist muscles frequently in a task-specific context. In the general population it is most common as writer's cramp, however, prevalence in pianist is relatively high with about 1–2% of pianists becoming affected. Involuntary flexion of fingers, abnormal muscular contractions (Figure 3A), and deterioration of fine motor control (Figure 3B) sometimes even terminate the professional career. The underlying pathophysiological mechanisms include anatomical and functional abnormalities in cortical and subcortical regions such as premotor areas, basal ganglia, and the cerebellum (Elbert et al., 1998; Kadota et al., 2010; Granert et al., 2011; Walter et al., 2012). It has been demonstrated that lack of surround inhibition and defective sensorimotor integration most probably cause the lack of specificity of motor commands in pianist's dystonia [for a review see Altenmüller (2003)]. Behaviorally, pianists with focal dystonia display a loss of timing accuracy of keystrokes during playing scales or other successions of piano tones (Jabusch et al., 2004; Rosenkranz et al., 2009). A decrease of accuracy of the individuated finger movements in pianists with focal dystonia further suggests a loss of independent control of finger movements. Furthermore, a neurophysiological study using TMS also demonstrated that force production of a muscle to move a particular finger facilitated not only adjacent muscles but also remote muscles, thus demonstrating the degradation of surround inhibition as a possible mechanism of loss of independent control of fingers (Rosenkranz et al., 2005). A preliminary study that investigated hand kinematics during playing a musical scale revealed that the symptomatic exaggerated flexion in a pianist with focal dystonia became more pronounced at faster tempi (Furuya and Altenmüller, 2012) (Figure 3C). Finally, the repetitive use of a particular combination of muscles in the hand yielded a loss of surround inhibition (Kang et al., 2012), which suggests that extensive training of the individuated finger movements may represent the two-sides of one coin that elicits virtuosity and focal dystonia (Rosenkranz et al., 2008).

## Conclusion

We have highlighted movement features specific to skilled piano performance. These unique motor skills can be associated with idiosyncratic demands of the various, highly elaborated motor tasks. For example, repetitive motion of the arm and hand for a prolonged time period, -uncommon in daily life motor tasks,—can lead to movement reorganization that facilitates physiological efficiency. Similarly, spatio-temporal constraints on motions of the individual fingers, which are specific to musical performance, can necessitate individuated finger movements. In this way, long-term piano training endows pianists with specialized organizations of redundant DOFs in arm and hand movements in piano playing. This, in turn facilitates speed, accuracy, and efficiency of the dexterous motor actions. Differences in movement organization between skilled pianists, unskilled pianists, and pianists with focal dystonia suggest that complex interactions between neuroplasticity and redundancy in the motor system via extensive piano practice yield skillful, but also disordered motor behaviors. It is therefore of importance to identify factors that influence this interaction in future studies, which should include motor learning experiments. For example, a paradigm that assesses intramanual and intermanual transfer effects of practice (Koeneke et al., 2009) could be applied to determine independence across different body parts in healthy and disordered pianists. In addition, a comparative approach across different musical instrumentalists would provide further insights into training-dependent characteristics of human neuroplasticity. For example, TMS-evoked finger movements in the left hand were more complex for violinists than pianists, indicating that neuroplastic changes reflect training history (Gentner et al., 2010). Training dependent neuroplasticity would also yield different organization of arm movements when comparing pianists, violinists, and cellists (Furuya and Kinoshita, 2007; Konczak et al., 2009; Verrel et al., 2013). By contrast, efficient muscular force production observed for both pianists and drummers (Fujii et al., 2009; Fujii and Moritani, 2012a,b) rather strengthens the idea that prolonged repetitive motions elicit neuroplastic changes that economize movements. Finally, to elaborate the understanding of control principles behind complex motor behaviors in piano playing, a computational approach that compares prediction of modeling with observed movements would be necessary (Kawato, 1999; Shadmehr and Krakauer, 2008; Kalveram and Seyfarth, 2009).

A significant implication of studies of behavioral movement science will aid musicians both in acquisition of complex motor skill efficiently and in prevention of playing-related neuromuscular disorders such as focal dystonia (Furuya et al., 2006; Altenmüller and Kopiez, 2010; Altenmüller et al., 2012). The evidence-based music pedagogy would enable musicians to accomplish more artistic and virtuosic musical performance, whereas the prevention can be a clue to resolve difficulty of the treatment of musicians' disorders.

### Conflict of interest statement

The authors declare that the research was conducted in the absence of any commercial or financial relationships that could be construed as a potential conflict of interest.
